# The Influence of *TEP1* and *TERC* Genetic Variants on the Susceptibility to Multiple Sclerosis

**DOI:** 10.3390/jcm12185863

**Published:** 2023-09-09

**Authors:** Gintarė Rumšaitė, Greta Gedvilaitė, Renata Balnytė, Loresa Kriaučiūnienė, Rasa Liutkevičienė

**Affiliations:** 1 Medical Faculty, Lithuanian University of Health Sciences, LT-50161 Kaunas, Lithuania; gintare.rumsaite@stud.lsmu.lt; 2 Neurosciences Institute, Medical Academy, Lithuanian University of Health Sciences, LT-50161 Kaunas, Lithuania; loresa.kriauciuniene@lsmuni.lt (L.K.); rasa.liutkeviciene@lsmuni.lt (R.L.); 3 Department of Neurology, Medical Academy, Lithuanian University of Health Sciences, LT-50161 Kaunas, Lithuania; renata.balnyte@lsmuni.lt

**Keywords:** multiple sclerosis, *TEP1* rs1760904, rs1713418, *TERC* rs12696304, rs35073794

## Abstract

Multiple sclerosis (MS) is a chronic inflammatory autoimmune disease of the central nervous system. According to recent studies, cellular senescence caused by telomere shortening may contribute to the development of MS. Aim of the study: Our aim was to determine the associations of *TEP1* rs1760904, rs1713418, *TERC* rs12696304, rs35073794 gene polymorphisms with the occurrence of MS. Methods: The study included 200 patients with MS and 230 healthy controls. Genotyping of *TEP1* rs1760904, rs1713418 and *TERC* rs12696304, rs35073794 was performed using RT-PCR. The obtained data were analysed using the program “IBM SPSS Statistics 29.0”. Haplotype analysis was performed using the online program “SNPStats”. Results: The *TERC* rs12696304 G allele of this SNP is associated with 1.4-fold lower odds of developing MS (*p* = 0.035). *TERC* rs35073794 is associated with approximately 2.4-fold reduced odds of MS occurrence in the codominant, dominant, overdominant, and additive models (*p* < 0.001; *p* < 0.001; *p* < 0.001; *p* < 0.001, respectively). Haplotype analysis shows that the rs1760904-G—rs1713418-A haplotype is statistically significantly associated with 1.75-fold increased odds of developing MS (*p* = 0.006). The rs12696304-C–rs35073794-A haplotype is statistically significantly associated with twofold decreased odds of developing MS (*p* = 0.008). In addition, the rs12696304-G—rs35073794-A haplotype was found to be statistically significantly associated with 5.3-fold decreased odds of developing MS (*p* < 0.001). Conclusion: The current evidence may suggest a protective role of *TERC* SNP in the occurrence of MS, while *TEP1* has the opposite effect.

## 1. Introduction

Multiple sclerosis (MS) is a chronic inflammatory autoimmune disease of the central nervous system (CNS) [1]. The incidence and prevalence rates of this disease are increasing worldwide. According to the Atlas of MS, 3rd edition, prepared by the Multiple Sclerosis International Federation, approximately 2.8 million people worldwide have MS. Lithuania belongs to the region of high prevalence and morbidity of MS (prevalence per 100,000 people is 101–200) [2]. MS is most commonly diagnosed in people between 20 and 40 years of age. Less commonly, it occurs in childhood (less than 1%) and after the age of 50 (about 2–10%) [3]. Moreover, MS is 2 to 3 times more common in women than in men [4]. MS is a multifactorial disease whose development is influenced by genetic and environmental factors [5]. Several pathological processes contribute to the development of MS, including blood–brain barrier (BBB) damage, multifocal inflammatory responses, demyelination, oligodendrocyte death, reactivated gliosis, and axonal degeneration [6]. 

According to recent studies, cellular senescence caused by telomere shortening may contribute to the development of MS [5]. To maintain the integrity of the genome, telomeres protect chromosomes from end fusion and degradation by exonucleases. Telomeres are specialized structures located at the ends of eukaryotic chromosomes, and are composed of tandem nucleotide repeats (TTAGGG) and proteins [7]. When telomeric DNA regions are critically shortened, they can signal replicative senescence of somatic cells and chromosome instability [8]. Telomerase is a ribonucleoprotein enzyme critical for replicating telomeric sequences in chromosomal DNA. The enzyme complex includes several components, including the telomerase RNA component (TERC), telomerase reverse transcriptase (TERT), dyskerin, and other accessory proteins such as TEP1 [8,9].

TERC binds to the 3′ end of chromosomes and provides a template sequence for reverse transcription catalysed by TERT [10]. According to recent studies, TERC inhibits apoptosis in immune cells, protects neurons from oxidative stress, and enhances cellular inflammatory responses [11]. TERC has been shown to increase the expression and release of inflammatory cytokines by directly binding to the promoters of the *LIN37*, *TPRG1L*, *TYROBP*, and *USP16* genes. These four genes encode proteins involved in the activation of the transcription factor nuclear factor κB (NF-κB) [11,12]. Inflammatory responses lead to the progressive shortening of telomeres, which has been linked to the development of age-related diseases [13]. It has been observed that naive CD4^+^ T cells from patients with rheumatoid arthritis exhibit increased telomerase inhibition, resulting in shortened telomeres due to decreased expression of TERT and TERC. For this reason, T cell subset aging is accelerated, and autoimmunity is activated [14]. TERC levels have been shown to be increased in individuals with MS or type II diabetes. It should be noted that these two diseases are associated with inflammatory responses [12].

The *TERC* gene is located on the long arm of chromosome 3 at position 26.2 (3q26.2) [15]. *TERC* is responsible for regulating telomere length [16]. Studies in mice have shown that *TERC* is involved in neural progenitor cell (NPC) proliferation. It has been observed that, in mice in which the *TERC* gene is knocked out, there is a statistically significant decrease in the proliferation of NPC. It has also been found that neurons cannot fully mature when the *TERC* gene is knocked out in NPC [17]. In addition, studies have shown that *TERC* gene polymorphisms influence the development of Alzheimer’s disease [18]. Since a relationship between *TERC* and MS has been established in the scientific literature, we decided to analyse the SNP rs12696304 and rs35073794 of the *TERC* gene. Polymorphisms in the *TERC* gene have been associated with changes in telomere length due to altered telomerase activity [19,20,21]. The SNP rs12696304 C > G is located in the downstream region of the *TERC* gene, i.e., 1.5 kb away from the transcription start nucleotide [21]. The rs35073794 A > G SNP is also located in the downstream region of the *TERC* gene [20]. Thus, polymorphisms in the *TERC* gene may promote cellular senescence by altering the stability of the telomerase complex or directly affecting the enzymatic activity of telomerase [18].

*TEP1* is responsible for RNA and protein binding and is involved in the regulation of telomere length [22,23]. *TEP1* is thought to function as a structural protein by binding to *TERC* and acting as a regulatory subunit to mediate the interaction of telomerase with other molecules [24]. In addition, *TEP1* and dyskerin are responsible for stabilizing the structure of telomerase [25]. In addition, *TEP1* directly interacts with the BLM protein of Bloom syndrome and regulates its helicase activity. Thus, it can be assumed that *TEP1* is involved in telomere lengthening [26].

The *TEP1* gene is located on the long arm of chromosome 14 at position 11.2 (14q11.2) [27]. According to NCBI, the *TEP1* gene consists of 55 exons [28]. This gene is responsible for telomere elongation and prevents neuron development due to DNA damage. Ren et al. found that *TEP1* is associated with white matter microstructure abnormality in schizophrenia [29]. Using whole-exome sequencing, Sebate and colleagues discovered that pathogenic mutations in the *TEP1* gene contribute to the neurodegenerative disease known as Parkinson’s [30]. According to the available data, there have been no studies investigating the association between *TEP1* and MS. Based on previous studies on neurological disorders, it can be assumed that the *TEP1* gene is involved in MS. In this study, we aimed to determine the influence of the *TEP1* gene SNP rs1760904 and rs1713418 on the occurrence of MS. The SNP rs1760904 A > G is located in the exon region of the *TEP1* gene [23,26]. Rs1760904 is a nonsynonymous SNP that causes a proline-to-serine substitution (Ser1195Pro) that may affect the *TEP1* structure and telomerase [26]. Rs1713418 A > G SNP is located in the 3′UTR region of the *TEP1* gene [26]. SNP in the 3′UTR alters the ability of miRNA to bind to the target gene, which affects gene regulation and increases the risk of MS [31].

The selected polymorphisms were chosen based on their potential relevance to our research topic. These specific genetic variants have previously been associated with biological processes related to telomere length in various studies [18,19,20,21]. By investigating the selected SNPs (rs1760904, rs1713418, rs12696304, and rs35073794), we aim to explore their potential contributions to the occurrence of multiple sclerosis. As mentioned above, each polymorphism is located within genes or regions that play crucial roles in telomere length regulation. As such, variations in these genetic loci may have functional consequences that interest us for our study. Therefore, this study aimed to determine the associations of *TEP1* rs1760904, rs1713418 and *TERC* rs12696304 and rs35073794 polymorphisms with occurrence in MS patients.

## 2. Materials and Methods

The study was performed at the Department of Neurology, Lithuanian University of Health Sciences and in the Ophthalmology laboratory, Neuroscience Institute, Lithuanian University of Health Sciences. Ethical approval for this study was obtained from the Kaunas Regional Biomedical Research Ethics Committee (No. BE-2-102, issued 14 November 2019). Each study participant signed the informed consent form. The subjects were divided into two groups:

1. The first group of study participants consisted of 200 MS patients (n = 200) aged 21 to 69 years. This group consisted of 98 (49%) female and 102 (51%) male participants. The MS diagnoses were confirmed using the 2017 diagnostic criteria: by clinical symptoms/relapses; magnetic resonance imaging (MRI) findings of the brain and/or spinal cord with typical demyelinating lesions (according to MAGNIMS criteria); and positive oligoclonal bands (OCBs) in cerebrospinal fluid (CSF) [32,33].
2. The second group of study participants consisted of 230 healthy volunteers (n = 230) aged 19 to 69 years. This group was composed of 133 (57.8%) females and 97 (42.2%) males. The control group consisted of individuals in good general health.

Patients were excluded if they had other systemic illnesses (diabetes mellitus, oncological diseases, systemic tissue disorders, chronic infectious diseases, autoimmune diseases, conditions after organ or tissue transplantation), eye optic system obscuration, or poor fundus photography quality. 

The demographic factors of the patients in the study’s MS group and the control group, i.e., age and gender, were evaluated in this study. The subjects were divided into <44 years old and ≥44 years old.

### 2.1. DNA Extraction and Genotyping

Genomic DNA was extracted from peripheral blood leukocytes by a salting-out method. Genotyping of *TEP1* rs1760904, rs1713418 and *TERC* rs12696304, rs35073794 was performed by real-time polymerase chain reaction (RT-PCR). To determine SNPs, we used TaqMan^®^ genotyping assays (Applied Biosystems, New York, NY, USA; Thermo Fisher Scientific, Inc., Waltham, MA, USA) according to the manufacturer’s recommendations. The assay IDs were C___1772362_20 (*TEP1* rs1760904), C___8921332_10 (*TEP1* rs1713418), C____407063_10 (*TERC* rs12696304), and C__58097851_10 (*TERC* rs35073794).

### 2.2. Statistical Analysis

The statistical analysis of the scientific work was carried out using “IBM SPSS Statistics 29.0.”. This study used Kolmogorov–Smirnov and Shapiro–Wilk tests to evaluate the hypothesis regarding the normal distribution of the measured trait values. Because the subjects’ characteristics did not meet the requirements of a normal distribution, the following descriptive statistics were used: median and interquartile range (IQR).

The χ^2^-test and Fisher’s exact test were used to compare the homogeneity of the genotypes and allele distributions of the *TEP1* rs1760904, rs1713418, *TERC* rs12696304, and rs35073794 gene polymorphisms. In addition, binary logistic regression was performed to evaluate the influence of genotypes and alleles on the occurrence of MS. Considering inheritance models and genotype combinations, the odds ratio (OR) was determined with a 95% confidence interval (CI). According to the Akaike Information Criterion (AIC), the model with the lowest value is the most appropriate inheritance model. As part of the analysis, the program “SNPStats” was also used to analyse the haplotypes. An evaluation of the linkage disequilibrium between the studied gene polymorphisms was performed. The deviation between the expected haplotype frequency and the observed frequency (D’) was calculated, and the square of the correlation coefficient of the haplotype frequency (r^2^) was evaluated. 

## 3. Results

The study involved 430 subjects who were divided into two groups: a control group (n = 230) and a group of subjects with MS (n = 200). After the formation of the study groups, genotyping of the *TEP1* rs1760904, rs1713418, *TERC* rs12696304, and rs35073794 polymorphisms was performed. The group of patients with MS consisted of 200 individuals: 98 females (49%) and 102 males (51%). The average age of the MS patients was 38 years. The control group consisted of 230 subjects: 133 females (57.8%) and 97 males (42.2%). The median age of the control group was 43.5 years. Sex and age did not differ between the groups. The demographic data of the subjects are shown in Table 1.

### 3.1. Associations of TEP1 (rs1760904, rs1713418) and TERC (rs12696304, rs35073794) with Multiple Sclerosis

The analysis of the genotype and allele distribution of *TEP1* rs1760904, rs1713418 and *TERC* rs12696304, rs35073794 revealed that the *TERC* rs12696304 G allele was less frequent in the MS group than in the control group (20.5% vs. 26.5%, *p* = 0.038). In addition, the *TERC* rs35073794 GG genotype was found to be more frequent in the MS group than in the control group (54.0% vs. 32.6%, *p* < 0.001). The AG Genotype and the A allele of the same polymorphism were less frequent in the MS group than in the control group (45.5% vs. 67.4%, *p* < 0.001; 23.25 vs. 33.7, *p* < 0.001) (Table 2).

No statistically significant differences were found in the distribution of genotypes and alleles of the *TEP1* gene rs1760904 and rs1713418 between the MS and control groups (Table 2).

After performing binary logistic regression, we found that each G allele of the *TERC* gene polymorphism rs12696304 was associated with a 1.4-fold decrease in the probability of occurrence of MS (OR: 0.703, (95% CI: 0.506–0.976), *p* = 0.035). *TERC* rs35073794 was associated with approximately 2.4-fold reduced odds of MS occurrence in the codominant, dominant, overdominant, and additive models (OR: 0.408, (95% CI: 0.275–0.603), *p* < 0.001; OR: 0.412 (95% CI: 0.279–0.610), *p* < 0.001; OR: 0.404 (95% CI: 0.273–0.598), *p* < 0.001; OR: 0.427 (95% CI: 0.289–0.629), *p* < 0.001, respectively) (Table 3). However, analysis of *TEP1* rs1760904 and rs1713418 revealed no statistically significant differences (Table 3).

### 3.2. Association of Single-Nucleotide Polymorphisms of TEP1 (rs1760904, rs1713418) and TERC (rs12696304, rs35073794) Genes with Multiple Sclerosis Regarding the Gender of the Subjects

Our analysis of SNP data in both males and females revealed that the *TERC* rs12696304 genotype CC and the C allele were more prevalent in the group of males with MS than in the control group of males (68.6% vs. 52.6%, *p* = 0.020; 83.33% vs. 72.16%, *p* = 0.007, respectively). In contrast, the *TERC* rs12696304 GG genotype was less frequent in the group of men with MS than in the control group of men (2.0% vs. 8.2%, *p* = 0.043). The TERC rs35073794 GG genotype was more frequent in the group of men with MS than in the control group of men (59.8% vs. 25.8%, *p* < 0.001). In addition, the AG genotype and the A allele of the same polymorphism were less frequent in the group of men with MS than in the control group of men (39.2% vs. 74.2%, *p* < 0.001; 20.59% vs. 37.11%, *p* < 0.001, respectively). No differences were found when the distribution of TEP1 rs1760904 and rs1713418 genotypes and alleles was analysed between men with MS and healthy men (Table 4).

No differences were found when comparing the distribution of genotypes and alleles of *TEP1* rs1760904, rs1713418 and *TERC* rs12696304, rs35073794 between women with MS and the female control group (*p* > 0.05) (Table 4).

Using binary logistic regression, we examined the effects of *TEP1* rs1760904, rs1713418 and *TERC* rs12696304, rs35073794 on the occurrence of MS in men and women separately.

When the male group was analysed, *TERC* rs12696304 was found to be associated with a decreased odds of MS occurrence in males in the codominant, dominant, and additive models (5.5-fold (OR: 0.182, (95% CI: 0.037–0.894), *p* = 0.036), 2-fold (OR = 0.507, (95% CI: 0.284–0.903), *p* = 0.021), and 1.9-fold (OR: 0.515, (95% CI: 0.314–0.845), *p* = 0.009), respectively). *TERC* rs35073794 was associated with approximately 4.4-fold decreased odds of MS occurring in males in the codominant, dominant, and overdominant models (OR: 0.228, (95% CI: 0.124–0.417), *p* < 0.001; OR: 0.233 (95% CI: 0.128–0.427), *p* < 0.001; OR: 0.224 (95% CI: 0.122–0.410), *p* < 0.001, respectively). In addition, we found that for *TERC* rs35073794, each A allele was associated with 3.9-fold decreased odds of MS occurrence in males (OR: 0.256, (95% CI: 0.141–0.462), *p* < 0.001). After performing binary logistic regression of *TEP1* rs1760904, rs1713418, we found no statistically significant differences between MS men and control men.

When binary logistic regression analysis of the *TEP1* rs1760904, rs1713418 and *TERC* rs12696304, rs35073794 gene polymorphisms was performed, no statistically significant differences were found between women with MS and healthy women (*p* > 0.05). The data are shown in Table 5.

### 3.3. Association of Single-Nucleotide Polymorphisms of the TEP1 (rs1760904, rs1713418) and TERC (rs12696304, rs35073794) Genes with Multiple Sclerosis Regarding the Age of the Subjects

In this study, we investigated the influence of the *TEP1* rs1760904, rs1713418 and *TERC* rs12696304, rs35073794 gene polymorphisms on the occurrence of MS according to age groups. The subjects were divided into < 44 years and ≥44 years old.

Genotype and allele distribution analysis revealed that the *TERC* rs35073794 GG genotype was more prevalent in MS patients younger than 44 years than in the control group (55.6% vs. 27.0%, *p* < 0.001). In addition, the same polymorphism A allele was found to be less frequent in MS patients younger than 44 years compared to the control group (22.2% vs. 36.5%, *p* < 0.001). When analysing the genotype and allele distribution of *TEP1* rs1760904, rs1713418 and *TERC* rs12696304, no statistically significant differences were found between MS patients younger than 44 years and the control group (*p* > 0.05). When comparing the distribution of genotypes and alleles of *TEP1* rs1760904, rs1713418 and *TERC* rs12696304, rs35073794, we found no statistically significant differences between subjects older than 44 years with MS and those in the control group (Table 6).

Based on the binary logistic regression of gene polymorphisms *TEP1* rs1760904, rs1713418, and *TERC* rs12696304, individuals younger than 44 years with MS and the control group showed no statistically significant differences. However, *TERC* rs35073794 was associated with an approximately 3.4-fold decrease in the odds of individuals younger than 44 years developing MS according to the dominant, overdominant, and additive models (OR: 0.295, (95% CI: 0.173–0.503), *p* < 0.001; OR: 0.295 (95% CI: 0.173–0.503), *p* < 0.001; OR: 0.295 (95% CI: 0.173–0.503), *p* < 0.001, respectively) (Table 7).

Binary logistic regression analysis in individuals over 44 years of age revealed that the *TEP1* rs1713418 GG genotype was associated with 2.2-fold increased odds of MS occurrence in individuals over 44 years of age compared with the AG and AA genotypes (OR: 2.191, (95% CI: 1.020–4.708), *p* = 0.044). However, our analysis of polymorphisms of the *TEP1* rs1760904 and TERC rs12696304, rs35073794 genes between the group of individuals with MS aged ≥44 years and the control group did not reveal statistically significant differences (Table 7).

### 3.4. Haplotype Analysis of TEP1 (rs1760904, rs1713418) and TERC (rs12696304, rs35073794)

The deviation between the expected haplotype and the observed frequency (D’) was equal to 0.471. It was also determined that the square of the correlation coefficient of haplotype frequency (r2) was 0.146 (Table 8). 

The *TEP1* rs1760904-A—rs1713418-A haplotype was the most common in the study group and was, therefore, selected as a reference. Haplotype analysis revealed that the rs1760904-G—rs1713418-A haplotype was associated with 1.7-fold increased odds of the occurrence of MS (OR: 1.74, (95% CI: 1.18–2.56), *p* = 0.006). In addition, the rs1760904-A—rs1713418-G haplotype was associated with 1.9-fold increased odds of the MS occurrence (OR: 1.92, (95% CI: 1.14–3.24), *p* = 0.014). The data are presented in Table 9.

According to our calculations, the deviation between the expected haplotype and the observed frequency (D’) was equal to 0.019. Moreover, the square of the haplotype frequency correlation coefficient (r2) was found to be <0.001 (Table 10). 

The *TERC* rs12696304-C—rs35073794-G haplotype was the most common in the study group and was, therefore, selected as a reference. Haplotype analysis revealed that the rs12696304-C—rs35073794-A haplotype was associated with a twofold reduction in the odds of MS occurrence (OR: 0.51, (95% CI: 0.32–0.84), *p* = 0.008). In addition, the rs12696304-G—rs35073794-A haplotype was associated with a 5.3-fold reduction in the odds of MS occurrence (OR: 0.19, (95% CI: 0.08–0.49), *p* < 0.001) (Table 11).

## 4. Discussion

In our study, we analysed the polymorphisms of the *TEP1* gene rs1760904, rs1713418, and the *TERC* gene rs12696304 and rs35073794 in 200 MS patients and 230 healthy individuals, because the SNPs we selected have not been studied in scientific research on the pathogenesis and development of MS. It should be noted that aging and genetic variants affecting telomere length, telomerase activation, and telomeric protein configuration can cause functional changes in cells [26,34]. Bühring, along with co-authors, prepared a meta-analysis which found seven studies on telomere length in MS indicating shorter telomeres in MS patients compared to controls, linked to increased disability and disease progression. This suggests a connection between aging, inflammation, and MS. TL assessment may be a disease progression biomarker. However, further research, including cell-specific analysis, is needed in order to understand MS’s pathophysiology and fully develop targeted therapies [35]. 

Sipos and co-authors found that TEP1 expression increases in ulcerative colitis during mild inflammation [36]. Gu and co-authors found that *TEP1* rs1713418 AG + AA genotypes were associated with 1.3-fold increased odds of prostate cancer in individuals younger than 69 years compared with the AA genotype (OR: 1.32, (95% CI: 1.02–1.70), *p* = 0.034). However, the AG + GG genotype of the same polymorphism was associated with 1.4-fold lower odds of prostate cancer in individuals older than >69 years compared to the AA genotype (OR: 0.71, (95% CI: 0.55–0.92), *p* = 0.010) [26]. Sun et al. found that *TEP1* rs1713418 was associated with 1.3-fold increased odds of ovarian cancer occurrence (OR: 1.33, (95% CI: 1.08–1.65), *p* = 0.009) [37]. It should be noted that excessive or persistent inflammation contributes to carcinogenesis and tumour progression through the activation of inflammatory molecules and signals [38]. Our study found that the *TEP1* rs1713418 GG genotype was associated with 2.2-fold increased odds of MS occurrence in individuals older than 44 years compared with the AG and AA genotypes (OR: 2.191, (95% CI: 1.020–4.708), *p* = 0.044).

Chan and co-authors performed a haplotype analysis and found that the *TEP1* haplotype, consisting of the SNP allele variants rs1713418, rs2104978, rs17211355, rs2297615, rs2228041, rs2228026, and rs1713440, was associated with 2.2-fold increased odds of bladder cancer occurrence (OR: 2.23, (95% CI: 1.13–4.60), *p* = 0.022) [39]. This study revealed that chronic inflammation may play a role in the development of malignancies, including bladder cancer [38]. Our haplotype analysis revealed that the rs1760904-G—rs1713418-A haplotype was statistically significantly associated with a 1.7-fold increased likelihood of developing MS (OR: 1.740, (95% CI: 1.180–2.560), *p* = 0.006). The rs1760904-A—rs1713418-G haplotype was statistically significantly associated with a 1.9-fold increased probability of the occurrence of MS (OR: 1.920, (95% CI: 1.140–3.240), *p* = 0.014).

Liu et al. found that TERC expression was increased more significantly in MS patients than in healthy individuals (*p* < 0.01) [12]. Scarabino and co-authors found that the *TERC* rs12696304 GG genotype correlated with the occurrence of Alzheimer’s disease [18]. In addition, the results of a study conducted by Sun and co-authors suggested that the *TERC* gene rs12696304 G allele and GG genotype were statistically significantly associated with 1.6-fold increased odds of developing chronic kidney disease (OR: 1.555, (95% CI: 1.215–1.990), *p* = 0.001; OR: 1.634, (95% CI: 1.201–2.234), *p* = 0.002, respectively). In addition, the researchers found that the G allele of the same polymorphism was associated with 1.8-fold increased odds of developing chronic kidney disease in the female group (OR:1.816, (95% CI: 1.248–2.641), *p* = 0.002), and the GG genotype was associated with 2-fold increased odds of developing chronic kidney disease in the female group (OR: 1.959, (95% CI: 1.233–3.114), *p* = 0.006). The authors also found that the rs12696304 G allele could contribute to a host autoimmune response targeting glomerular tissues by activating the NF-κB pathway via TERC [14]. It is known that secretory renal dysfunction (decreased synthesis of vitamin D, erythropoietin, and Klotho protein) may contribute to brain dysfunction in MS patients [40]. According to the results of Al Khaldu and co-authors, the genotype of *TERC* gene rs12696304 GG was associated with a 1.6-fold increased probability of developing type 2 diabetes (OR: 1.6, (95% CI: 1.5–1.9), *p* = 0.005) [41]. It should be noted that diabetes, like MS, is associated with increased oxidative stress and inflammatory responses, which may accelerate telomere shortening and associated cellular senescence [42]. 

After performing binary logistic regression, we found that the *TERC* gene rs12696304 G allele was associated with a 1.4-fold decrease in the likelihood of the occurrence of MS (OR: 0.703, (95% CI: 0.506–0.976), *p* = 0.035). *TERC* rs12696304 was associated with a decreased probability of the occurrence of MS in males according to the codominant, dominant, and additive models (5.5-fold (OR: 0.182, (95% CI: 0.037–0.894), *p* = 0.036), 2-fold (OR =0.507, (95% CI: 0.284–0.903), *p* = 0.021), and 1.9-fold (OR: 0.515, (95% CI: 0.314–0.845), *p* = 0.009). However, we found no statistically significant associations between these polymorphisms and MS risk in females. It is well-known that genetic factors contributing to disease susceptibility can vary between genders [43]. This phenomenon can arise due to hormonal differences or gender-specific gene expression. Hormones such as oestrogen and testosterone can influence immune responses, interact with genetic factors, and might affect the risk of autoimmune diseases like MS [44].

Wu and co-authors found that *TERC* rs35073794 was associated with 2.4-fold increased odds of renal cell carcinoma (RCC) occurrence in an allele model (A/G) (OR =2.39, 95% CI = 0.99–5.80, *p* = 0.047). The authors also found that the rs35073794 AG genotype is associated with a 2.6-fold increased odds of RCC risk with adjustment for gender, age and BMI (OR =2.61, 95% CI = 1.01–6.76, *p* = 0.045) [20]. We found that *TERC* rs35073794 is associated with about 2.4-fold decreased odds of MS development under the codominant, dominant, overdominant, and additive models (OR: 0.408, (95% CI: 0.275–0.603), *p* < 0.001; OR: 0.412 (95% CI: 0.279–0.610), *p* < 0.001; OR: 0.404 (95% CI: 0.273–0.598), *p* < 0.001; OR: 0.427 (95% CI: 0.289–0.629), *p* < 0.001, respectively). *TERC* rs35073794 is associated with about 4.4-fold decreased odds of MS occurrence in men according to the codominant, dominant, and overdominant models (OR: 0.228, (95% CI: 0.124–0.417), *p* < 0.001; OR: 0.233 (95% CI: 0.128–0.427), *p* < 0.001; OR: 0.224 (95% CI: 0.122–0.410), *p* < 0.001, respectively). Furthermore, for *TERC* rs35073794, each A allele was associated with 3.9-fold decreased odds of MS occurrence (OR: 0.256, (95% CI: 0.141–0.462), *p* < 0.001). *TERC* rs35073794 was associated with about 3.4-fold decreased odds of subjects younger than 44 years of age developing MS according to the dominant, overdominant, and additive models (OR: 0.295, (95% CI: 0.173–0.503), *p* < 0.001; OR: 0.295 (95% CI: 0.173–0.503), *p* < 0.001; OR: 0.295 (95% CI: 0.173–0.503), *p* < 0.001, respectively). 

Based on haplotype analysis, Maubaret and colleagues found that the *TERC* rs12696304-G-rs10936601-T-rs16847897-C haplotype was statistically significantly associated with a 1.35-fold reduction in the risk of developing type 2 diabetes (OR: 0.74, (95% CI: 0.61–0.91), *p* = 0.004) [45]. According to our haplotype analysis, the rs12696304-C-rs35073794-A haplotype was associated with a twofold reduction in the likelihood of developing MS (OR: 0.51, (95% CI: 0.32–0.84), *p* = 0.008). In addition, we discovered that the rs12696304-G-rs35073794-A haplotype was associated with a 5.3-fold reduction in the probability of MS occurrence (OR: 0.19, (95% CI: 0.08–0.49), *p* < 0.001).

In our study, we acknowledge several limitations. It is important to note that genetic determinants of telomere length may exhibit variations across different racial and ethnic groups. Our study was conducted exclusively among Lithuanian participants, which may limit the generalizability of our findings to more diverse populations. Future research should consider including a more ethnically and racially diverse sample in order to better understand the potential variability in genetic associations with telomere length. Also, for more accurate results, the sample size should be increased. 

The study exhibits strengths in its rigorous methodology, which is characterized by clear objectives, appropriate sample sizes, and robust data collection. These elements significantly enhance the reliability and validity of our study’s findings. Additionally, the study adhered to standardized protocols and procedures, enabling the potential for replication and facilitating comparability with other studies.

There is evidence that telomere-related genes play a critical role in carcinogenesis. However, it is still unclear whether alterations in telomere-related genes may contribute to the progression and occurrence of MS [26]. Therefore, this study warrants further research to explain the pathogenesis of MS and the impact of telomere-related gene alterations on its development. 

## 5. Conclusions

The current evidence may suggest that *TERC* SNP plays a protective role in the occurrence of MS, whereas *TEP1* has the opposite effect, but research is still in the early stages, so it is premature to draw firm conclusions.

## Figures and Tables

**Table 1:** Demographic characteristics of the study groups.

Characteristics		Group		*p*-Value
		MS Group	Control Group	
Gender	Males, N (%)	102 (51)	97 (42.2)	0.067
	Females, N (%)	98 (49)	133 (57.8)	
Age, Median (IQR)		38 (15)	43.5 (28)	0.117

IQR—interquartile range; MS—multiple sclerosis; *p*—significance level.

**Table 2:** Distribution of the TEP1 rs1760904, rs1713418 and TERC rs12696304, rs35073794 genotypes and alleles in patients with MS and control group subjects.

Gene, SNP	Genotype and Allele	Distribution		
		MS Group, N (%)	Control Group, N (%)	*p*-Value
*TEP1* rs1760904	Genotype			0.219
	AA	44 (22.00)	56 (24.30)	
	AG	96 (48.00)	122 (53.00)	
	GG	60 (30.00)	52 (22.60)	
	Allele			0.154
	A	184 (46.00)	234 (50.87)	
	G	216 (54.00)	226 (49.13)	
*TEP1* rs1713418	Genotype			0.222
	AA	72 (36.00)	81 (35.20)	
	AG	86 (43.00)	114 (49.60)	
	GG	42 (21.00)	35 (15.20)	
	Allele			0.457
	A	230 (57.50)	276 (60.00)	
	G	170 (42.50)	184 (40.00)	
*TERC* rs12696304	Genotype			0.092
	CC	124 (62.00)	123 (53.50)	
	CG	70 (35.00)	92 (40.00)	
	GG	6 (3.00)	15 (6.50)	
	Allele			**0.038**
	C	318 (79.50)	338 (73.48)	
	G	82 (20.50)	122 (26.52)	
*TERC* rs35073794	Genotype			**<0.001**
	GG	108 (54.00) ^1^	75 (32.60) ^1^	
	AG	91 (45.50) ^2^	155 (67.40) ^2^	
	AA	1 (0.50)	0 (0.00)	
	Allele			**<0.001**
	G	307 (76.75)	305 (66.30)	
	A	93 (23.25)	155 (33.70)	

SNP—single-nucleotide polymorphism; MS—multiple sclerosis; *p*-value—significance level (statistically significant when *p* < 0.05). Statistically significant results are bold. ^1^ GG vs. AG + AA *p* < 0.001. ^2^ AG vs. GG + AA *p* < 0.001.

**Table 3:** Binary logistic regression analysis of TEP1 rs1760904, rs1713418 and TERC rs12696304, rs35073794 in patients with MS and control group subjects.

Model	Genotype/Allele	OR (95% CI)	*p*-Value	AIC
*TEP1* rs1760904				
Codominant	AG vs. AA	1.001 (0.622–1.613)	0.995	594.983
	GG vs. AA	1.469 (0.854–2.525)	0.165	
Dominant	AG + GG vs. AA	1.141 (0.727–1.790)	0.566	595.681
Recessive	GG vs. AG + AA	1.467 (0.952–2.260)	0.082	592.983
Overdominant	AG vs. AA + GG	0.817 (0.559–1.194)	0.297	594.923
Additive	G	1.220 (0.930–1.600)	0.152	593.947
*TEP1* rs1713418				
Codominant	AG vs. AA	0.849 (0.556–1.296)	0.447	595.007
	GG vs. AA	1.350 (0.779–2.339)	0.284	
Dominant	AG + GG vs. AA	0.966 (0.651–1.436)	0.866	595.983
Recessive	GG vs. AG + AA	1.481 (0.903–2.430)	0.120	593.584
Overdominant	AG vs. AA + GG	0.768 (0.524–1.124)	0.174	594.156
Additive	G	1.104 (0.845–1.443)	0.466	595.481
*TERC* rs12696304				
Codominant	CG vs. CC	0.755 (0.507–1.124)	0.166	593.121
	GG vs. CC	0.397 (0.149–1.056)	0.064	
Dominant	CG + GG vs. CC	0.705 (0.479–1.036)	0.075	592.826
Recessive	GG vs. CG + CC	0.443 (0.169–1.165)	0.099	593.043
Overdominant	CG vs. CC + GG	0.808 (0.546–1.196)	0.286	594.871
Additive	G	0.703 (0.506–0.976)	**0.035**	591.502
*TERC* rs35073794				
Codominant	AG vs. GG	0.408 (0.275–0.603)	**<0.001**	575.893
	AA vs. GG	-	-	
Dominant	AG + AA vs. GG	0.412 (0.279–0.610)	**<0.001**	575.875
Recessive	AA vs. AG + GG	-	-	-
Overdominant	AG vs. AA + GG	0.404 (0.273–0.598)	**<0.001**	574.944
Additive	A	0.427 (0.289–0.629	**<0.001**	577.123

OR—odds ratio; CI—confidence interval; *p*-value—significance level (statistically significant when *p* < 0.05). Statistically significant results are bold. AIC—Akaike information criterion.

**Table 4:** Distribution of TEP1 rs1760904, rs1713418 and TERC rs12696304, rs35073794 genotypes and alleles in patients with MS and control group subjects regarding gender.

Genotype and Allele	Males		*p*-Value	Females		*p*-Value
	MS Group, N (%)	Control Group, N (%)		MS Group, N (%)	Control Group, N (%)	
*TEP1* rs1760904						
Genotype			0.395			0.403
AA	25 (24.50)	28 (28.90)		19 (19.40)	28 (21.10)	
AG	50 (49.00)	51 (52.60)		46 (46.90)	71 (53.40)	
GG	27 (26.50)	18 (18.60)		33 (33.70)	34 (25.60)	
Allele			0.221			0.297
A	100 (49.02)	107 (55.15)		84 (42.86)	127 (47.74)	
G	104 (50.98)	87 (44.85)		112 (57.14)	139 (52.26)	
*TEP1* rs1713418						
Genotype			0.486			0.181
AA	40 (39.20)	32 (33.00)		32 (32.70)	49 (36.80)	
AG	46 (45.10)	52 (53.60)		40 (40.80)	62 (46.60)	
GG	16 (15.70)	13 (13.40)		26 (26.50)	22 (16.50)	
Allele			0.687			0.128
A	126 (61.76)	116 (59.79)		104 (53.06)	160 (60.15)	
G	78 (38.24)	78 (40.21)		92 (46.94)	106 (39.85)	
*TERC* rs12696304						
Genotype			**0.025**			0.916
CC	70 (68.60) ^1^	51 (52.60) ^1^		54 (55.10)	72 (54.10)	
CG	30 (29.40)	38 (39.20)		40 (40.80)	54 (40.60)	
GG	2 (2.00) ^2^	8 (8.20) ^2^		4 (4.10)	7 (5.30)	
Allele			**0.007**			0.792
C	170 (83.33)	140 (72.16)		148 (75.51)	198 (74.44)	
G	34 (16.67)	54 (27.84)		48 (24.49)	68 (25.56)	
*TERC* rs35073794						
Genotype			**<0.001**			0.115
GG	61 (59.80) ^3^	25 (25.80) ^3^		47 (48.00)	50 (37.60)	
AG	40 (39.20) ^4^	72 (74.20) ^4^		51 (52.00)	83 (62.40)	
AA	1 (1.00)	0 (0.00)				
Allele			**<0.001**			0.225
G	162 (79.41)	122 (62.89)		145 (73.98)	183 (68.80)	
A	42 (20.59)	72 (37.11)		51 (26.02)	83 (31.20)	

MS—multiple sclerosis; *p*-value—significance level (statistically significant when *p* < 0.05). Statistically significant results are bold. ^1^ CC vs. CG + GG *p* = 0.020. ^2^ GG vs. CG + CC *p* = 0.043. ^3^ GG vs. AG + AA *p* < 0.001. ^4^ AG vs. GG + AA *p* < 0.001.

**Table 5:** Binary logistic regression analysis of TEP1 rs1760904, rs1713418 and TERC rs12696304, rs35073794 in patients with MS and control group subjects regarding gender.

Model	Genotype/Allele	OR (95% CI)	*p*-Value	AIC
Males				
*TEP1* rs1760904				
Codominant	AG vs. AA	1.098 (0.564–2.136)	0.783	277.881
	GG vs. AA	1.680 (0.752–3.754)	0.206	
Dominant	AG + GG vs. AA	1.250 (0.666–2.346)	0.488	277.264
Recessive	GG vs. AG + AA	1.580 (0.805–3.103)	0.184	275.956
Overdominant	AG vs. AA + GG	0.867 (0.497–1.513)	0.616	277.495
Additive	G	1.286 (0.862–1.918)	0.218	276.218
*TEP1* rs1713418				
Codominant	AG vs. AA	0.708 (0.384–1.304)	0.267	278.303
	GG vs. AA	0.985 (0.414–2.343)	0.972	
Dominant	AG + GG vs. AA	0.763 (0.427–1.364)	0.361	276.911
Recessive	GG vs. AG + AA	1.202 (0.545–2.652)	0.648	277.538
Overdominant	AG vs. AA + GG	0.711 (0.407–1.242)	0.231	276.305
Additive	G	0.918 (0.609–1.383)	0.682	277.579
*TERC* rs12696304				
Codominant	CG vs. CC	0.575 (0.316–1.047)	0.071	272.078
	GG vs. CC	0.182 (0.037–0.894)	**0.036**	
Dominant	CG + GG vs. CC	0.507 (0.284–0.903)	**0.021**	272.350
Recessive	GG vs. CG + CC	0.223 (0.046–1.075)	0.062	273.377
Overdominant	CG vs. CC + GG	0.647 (0.359–1.167)	0.148	275.637
Additive	G	0.515 (0.314–0.845)	**0.009**	270.497
*TERC* rs35073794				
Codominant	AG vs. GG	0.228 (0.124–0.417)	**<0.001**	253.671
	AA vs. GG	-	-	
Dominant	AG + AA vs. GG	0.233 (0.128–0.427)	**<0.001**	253.714
Recessive	AA vs. AG + GG	-	-	-
Overdominant	AG vs. AA + GG	0.224 (0.122–0.410)	**<0.001**	252.353
Additive	A	0.256 (0.141–0.462)	**<0.001**	255.882
Females				
*TEP1* rs1760904				
Codominant	AG vs. AA	0.955 (0.479–1.905)	0.896	317.102
	GG vs. AA	1.430 (0.673–3.041)	0.352	
Dominant	AG + GG vs. AA	1.109 (0.578–2.127)	0.756	316.814
Recessive	GG vs. AG + AA	1.478 (0.834–2.619)	0.181	315.119
Overdominant	AG vs. AA + GG	0.772 (0.458–1.303)	0.333	315.972
Additive	G	1.224 (0.840–1.784)	0.293	315.798
*TEP1* rs1713418				
Codominant	AG vs. AA	0.988 (0.544–1.795)	0.968	315.523
	GG vs. AA	1.810 (0.879–3.724)	0.107	
Dominant	AG + GG vs. AA	1.203 (0.694–2.085)	0.510	316.474
Recessive	GG vs. AG + AA	1.822 (0.960–3.457)	0.066	313.525
Overdominant	AG vs. AA + GG	0.790 (0.466–1.339)	0.381	316.139
Additive	G	1.302 (0.911–1.862)	0.148	314.801
*TERC* rs12696304				
Codominant	CG vs. CC	0.988 (0.576–1.695)	0.964	318.732
	GG vs. CC	0.762 (0.212–2.735)	0.677	
Dominant	CG + GG vs. CC	0.962 (0.569–1.624)	0.884	316.889
Recessive	GG vs. CG + CC	0.766 (0.218–2.693)	0.678	316.734
Overdominant	CG vs. CC + GG	1.009 (0.593–1.716)	0.974	316.909
Additive	G	0.940 (0.602–1.466)	0.784	316.835
*TERC* rs35073794				
Codominant	AG vs. GG	-	-	-
	AA vs. GG	-	-	
Dominant	AG + AA vs. GG	0.654 (0.385–1.110)	0.115	314.425
Recessive	AA vs. AG + GG	-	-	-
Overdominant	AG vs. AA + GG	0.654 (0.385–1.110)	0.115	314.425
Additive	A	0.654 (0.385–1.110)	0.115	314.425

OR—odds ratio; CI—confidence interval; *p*-value—significance level (statistically significant when *p* < 0.05); statistically significant results are bold; AIC—Akaike information criterion.

**Table 6:** Distribution of the TEP1 rs1760904, rs1713418 and TERC rs12696304, rs35073794 genotypes and alleles in patients with MS and control group subjects regarding age.

Genotype and Allele	<44		*p*-Value	≥44		*p*-Value
	MS Group, N (%)	Control Group, N (%)		MS Group, N (%)	Control Group, N (%)	
*TEP1* rs1760904						
Genotype			0.509			0.620
AA	28 (20.70)	27 (23.50)		16 (24.60)	29 (25.20)	
AG	64 (47.40)	59 (51.30)		32 (49.20)	63 (54.80)	
GG	43 (31.90)	29 (25.20)		17 (26.20)	23 (20.00)	
Allele			0.295			0.538
A	120 (44.44)	113 (49.13)		64 (49.23)	121 (52.61)	
G	150 (55.56)	117 (50.87)		66 (50.77)	109 (47.39)	
*TEP1* rs1713418						
Genotype			0.603			0.119
AA	54 (40.00)	41 (35.70)		18 (27.70)	40 (34.80)	
AG	56 (41.50)	55 (47.80)		30 (46.20)	59 (51.30)	
GG	25 (18.50)	19 (16.50)		17 (26.20)	16 (13.90)	
Allele			0.789			0.075
A	164 (60.74)	137 (59.57)		66 (50.77)	139 (60.43)	
G	106 (39.26)	93 (40.43)		64 (49.23)	91 (39.57)	
*TERC* rs12696304						
Genotype			0.246			0.567
CC	87 (64.40)	63 (54.80)		37 (56.90)	60 (52.20)	
CG	45 (33.30)	47 (40.90)		25 (38.50)	45 (39.10)	
GG	3 (2.20)	5 (4.30)		3 (4.60)	10 (8.70)	
Allele			0.110			0.363
C	219 (81.11)	173 (75.22)		99 (76.15)	165 (71.74)	
G	51 (18.89)	57 (24.78)		31 (23.85)	65 (28.26)	
*TERC* rs35073794						
Genotype			**<0.001**			0.094
AA	60 (44.40)	84 (73.00)		1 (1.50)	0 (0.00)	
AG	75 (55.60)	31 (27.00)		31 (47.70)	71 (61.70)	
GG				33 (50.80)	44 (38.30)	
Allele			**<0.001**			0.270
A	60 (22.22)	84 (36.52)		33 (25.39)	71 (30.87)	
G	210 (77.78)	146 (63.48)		97 (74.61)	159 (69.13)	

MS—multiple sclerosis; *p*-value—significance level (statistically significant when *p* < 0.05). Statistically significant results are bold.

**Table 7:** Binary logistic regression analysis of TEP1 rs1760904, rs1713418 and TERC rs12696304, rs35073794 in patients with MS and control group subjects regarding age.

Model	Genotype/Allele	OR (95% CI)	*p*-Value	AIC
<44				
*TEP1* rs1760904				
Codominant	AG vs. AA	1.046 (0.554–1.976)	0.890	347.612
	GG vs. AA	1.430 (0.704–2.902)	0.322	
Dominant	AG + GG vs. AA	1.172 (0.644–2.135)	0.603	346.701
Recessive	GG vs. AG + AA	1.386 (0.796–2.415)	0.249	345.632
Overdominant	AG vs. AA + GG	0.856 (0.520–1.408)	0.539	346.594
Additive	G	1.205 (0.848–1.713)	0.299	345.887
*TEP1* rs1713418				
Codominant	AG vs. AA	0.773 (0.446–1.341)	0.360	347.959
	GG vs. AA	0.999 (0.486–2.056)	0.998	
Dominant	AG + GG vs. AA	0.831 (0.497–1.390)	0.480	346.473
Recessive	GG vs. AG + AA	1.148 (0.596–2.214)	0.680	346.801
Overdominant	AG vs. AA + GG	0.773 (0.469–1.276)	0.315	345.959
Additive	G	0.955 (0.675–1.351)	0.796	346.905
*TERC* rs12696304				
Codominant	CG vs. CC	0.693 (0.411–1.168)	0.169	346.168
	GG vs. CC	0.434 (0.100–1.885)	0.266	
Dominant	CG + GG vs. CC	0.668 (0.402–1.112)	0.121	344.557
Recessive	GG vs. CG + CC	0.500 (0.117–2.139)	0.350	346.065
Overdominant	CG vs. CC + GG	0.723 (0.432–1.212)	0.219	345.457
Additive	G	0.682 (0.435–1.071)	0.097	344.182
*TERC* rs35073794				
Codominant	AG vs. GG	-	-	-
	AA vs. GG	-	-	
Dominant	AG + AA vs. GG	0.295 (0.173–0.503)	**<0.001**	325.726
Recessive	AA vs. AG + GG	-	-	-
Overdominant	AG vs. AA + GG	0.295 (0.173–0.503)	**<0.001**	325.726
Additive	A	0.295 (0.173–0.503)	**<0.001**	325.726
≥44				
*TEP1* rs1760904				
Codominant	AG vs. AA	0.921 (0.437–1.937)	0.828	238.517
	GG vs. AA	1.340 (0.558–3.214)	0.512	
Dominant	AG + GG vs. AA	1.033 (0.511–2.088)	0.929	237.452
Recessive	GG vs. AG + AA	1.417 (0.691–2.903)	0.341	236.564
Overdominant	AG vs. AA + GG	0.800 (0.435–1.472)	0.474	236.946
Additive	G	1.154 (0.741–1.799)	0.526	237.057
*TEP1* rs1713418				
Codominant	AG vs. AA	1.130 (0.556–2.296)	0.736	235.321
	GG vs. AA	2.361 (0.979–5.696)	0.056	
Dominant	AG + GG vs. AA	1.393 (0.716–2.708)	0.329	236.492
Recessive	GG vs. AG + AA	2.191 (1.020–4.708)	**0.044**	233.436
Overdominant	AG vs. AA + GG	0.814 (0.442–1.497)	0.507	237.019
Additive	G	1.492 (0.959–2.320)	0.076	234.263
*TERC* rs12696304				
Codominant	CG vs. CC	0.901 (0.476–1.705)	0.748	238.256
	GG vs. CC	0.486 (0.126–1.884)	0.297	
Dominant	CG + GG vs. CC	0.826 (0.448–1.523)	0.539	237.082
Recessive	GG vs. CG + CC	0.508 (0.135–1.917)	0.318	236.359
Overdominant	CG vs. CC + GG	0.972 (0.521–1.815)	0.930	237.452
Additive	G	0.795 (0.485–1.305)	0.365	236.627
*TERC* rs35073794				
Codominant	AG vs. GG	0.582 (0.314–1.080)	0.086	234.455
	AA vs. GG	-	-	
Dominant	AG + AA vs. GG	0.601 (0.325–1.111)	0.104	234.814
Recessive	AA vs. AG + GG	-	-	-
Overdominant	AG vs. AA + GG	0.565 (0.305–1.045)	0.069	234.132
Additive	A	0.650 (0.356–1.190)	0.163	235.502

OR—odds ratio; CI—confidence interval; *p*-value—significance level (statistically significant when *p* < 0.05). Statistically significant results are bold. AIC—Akaike information criterion.

**Table 8:** Linkage disequilibrium between TEP1 rs1760904 and rs1713418 polymorphisms in patients with MS and the control group.

SNP	MS Group vs. Control Group		
	D’	r^2^	*p*-Value
rs1760904–rs1713418	0.471	0.146	0.000

SNP—single-nucleotide polymorphism; MS—multiple sclerosis; D’—the deviation between the expected and observed haplotype frequency; r^2^—the haplotype frequency correlation coefficient square; *p*-value—significance level (statistically significant when *p* < 0.05).

**Table 9:** Haplotype association with predisposition to MS occurrence.

Haplotype	*TEP1* rs1760904	*TEP1* rs1713418	Frequency, %		OR (95% CI)	*p*-Value
			Control	MS		
1	A	A	42.81	32.53	1.000	-
2	G	G	31.94	29.03	1.140 (0.820–1.600)	0.430
3	G	A	17.19	24.97	1.740 (1.180–2.560)	**0.006**
4	A	G	8.06	13.47	1.920 (1.140–3.240)	**0.014**

MS—multiple sclerosis; OR—odds ratio; CI—confidence interval; *p*-value—significance level (statistically significant when *p* < 0.05). Statistically significant results are bold.

**Table 10:** Linkage disequilibrium between the TERC rs12696304 and rs35073794 polymorphisms in patients with MS and the control group.

SNP	MS Group vs. Control Group		
	D’	r^2^	*p*-Value
rs12696304–rs35073794	0.019	<0.001	0.631

SNP—single-nucleotide polymorphism; MS—multiple sclerosis; D’—the deviation between the expected and observed haplotype frequency; r^2^—the haplotype frequency correlation coefficient square; *p*-value—significance level (statistically significant when *p* < 0.05).

**Table 11:** Haplotype association with the predisposition to MS occurrence.

Haplotype	*TERC* rs12696304	*TERC* rs35073794	Frequency, %		OR (95% CI)	*p*-Value
			Control	MS		
1	C	G	50.48	59.55	1.000	-
2	C	A	22.99	19.95	0.510 (0.320–0.840)	**0.008**
3	G	G	15.82	17.20	0.870 (0.540–1.390)	0.550
4	G	A	10.70	3.30	0.190 (0.080–0.490)	**<0.001**

MS—multiple sclerosis; OR—odds ratio; CI—confidence interval; *p*-value—significance level (statistically significant when *p* < 0.05); statistically significant results are bold.

## Data Availability

The data will be sent upon request.
